# Affected Children of Healthy Parents: Multiple Pediatric Cases of Autosomal Recessive Charcot-Marie-Tooth Disease in a Pakistani Family

**DOI:** 10.7759/cureus.4417

**Published:** 2019-04-09

**Authors:** Khushboo Nusrat, Samar Mahmood, Mohammad Raza, Shayan Marsia, Awais Abbas

**Affiliations:** 1 Internal Medicine, Dow University of Health Sciences (DUHS), Karachi, PAK; 2 Pediatrics, Dow University of Health Sciences (DUHS), Karachi, PAK

**Keywords:** neuropathy, peripheral nervous system, sural nerve biopsy, hereditary motor and sensory neuropathy, autosomal recessive, pakistan, charcot-marie-tooth, peripheral nervous system, pes cavus, pediatrics

## Abstract

Charcot-Marie-Tooth (CMT) disease, also referred to as hereditary motor and sensory neuropathy (HMSN), is a heterogeneous group of disorders which primarily affects the peripheral nervous system. Clinically, the main features are progressive muscle weakness seen distally, along with wasting seen predominantly in the anterior compartments of the lower legs. The disease can broadly be classified into two groups, CMT1 and CMT2-based on inheritance patterns, paired with anatomical or electrophysiological findings. It can be inherited in the autosomal dominant, X-linked and rarely, the autosomal recessive fashions. Here, we present an unusual case of autosomal recessive CMT disease, in four out of six children of unaffected parents in a family.

## Introduction

Charcot-Marie-Tooth (CMT) disease is a hereditary neuropathy, affecting both motor and sensory nerves. Discovered in 1886, the disease is named after its three identifiers. The classification of the condition is normally done based on inheritance patterns, paired with anatomical or electrophysiological findings of the disease. Broadly speaking, the disease can be classified into two groups, with CMT1 characterized by slow nerve conduction and hypertrophy of nerves owing to demyelination, and CMT2 characterized as normal or subnormal nerve conduction due to axonal degeneration [1].

Attempts to determine the prevalence of CMT have yielded in a range of estimates from different places in the world-from 9.7 per 100,000 in Serbia, to 82.3 per 100,000 in Norway and 22.5 per 100,000 in Denmark [2]. The prevalence of the disease subtype is known to vary by region as well, with CMT type 1A being most common in Western regions and the other forms more frequent in the Asian population [3]. Regarding the form of inheritance, the condition can be transmitted in an autosomal dominant, autosomal recessive, or X-linked fashion. The clinical features of the disease become apparent in the first to second decades of life [4].

Here, we present a case of CMT disease in four out of six children of unaffected parents, rare in the autosomal recessive nature of their condition as most familial cases are known to present in individuals with affected parents.

## Case presentation

Two brothers presented to us, both with similar symptoms. Our first patient was an eight-year-old male who presented with an inability to stand or walk since the past two months, along with bilateral foot deformities. According to his father, the patient had developed a difficulty in walking and in climbing stairs, accompanied by frequent falls - about six months back. Gradually, he had lost the ability to walk even with support and was mainly confined to his bed-although he could sit up and crawl.

The patient’s intelligence was unaffected by the illness; he had no history of trauma, fever, fits, incontinence, or syncope and did not display vision, speech, or hearing abnormalities. A detailed review of the gastro-intestinal, genitourinary, respiratory, and cardiovascular systems showed no abnormality. The patients’ parents were first cousins, albeit unaffected by the disease themselves. However, out of five siblings, two of the patient’s sisters (12 and 14 years of age) and one brother (five years old) were affected by a similar illness. The patient had had an unremarkable birth history, had reached all the relevant milestones timely and was said to be taking a nutritionally adequate diet. As per the parents, all his vaccinations were complete and the past medical history was clear.

On general examination, the patient was well oriented in time, place, and person with his vitals, height, and weight all within the normal ranges. Regarding system-wise examination, the central nervous system examination showed no signs of wasting or abnormal tone in the upper limbs, the power in both the upper limbs was 4/5, and the deep tendon reflexes were normal when elicited. However, the bulk of both the lower limbs was decreased, with the right lower limb being slightly more wasted than the left. The tone was decreased as well and power in both the lower limbs was 2/5. The deep tendon reflexes of the lower limbs were absent. On further examination, Babinski sign was negative and the pupils were bilaterally and equally, reactive to light. The gait of the patient could not be assessed as he could not stand. However, there were no signs pointing towards cerebellar or cranial nerve dysfunction and mental functions and speech proved to be intact. On sensory examination, a higher threshold to pain and temperature was noted.

**Figure 1:**
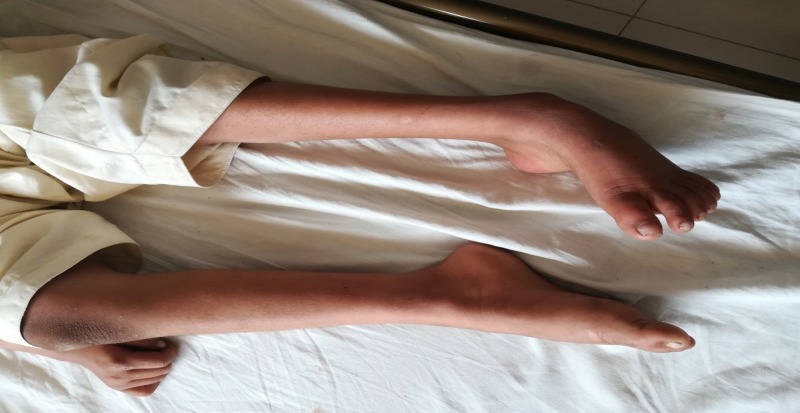
Bilateral foot drop with pes cavus in patient 1 - more pronounced on the left side.

On examination of the musculoskeletal system, the patient had marked wasting in the anterior compartments of both legs. He demonstrated a bilateral foot drop with pes cavus (Figure 1)-more pronounced on the left side - and his feet were kept in a plantar, fixed position in the relaxed state. Contractures on the knees and Achilles tendon were seen. The upper limbs did not show any marked abnormality other than contractures over the interphalangeal joints of the fingers, with the skin prominently thicker there. The examinations of all the other systems were unremarkable.

As per laboratory investigations: the complete blood count, electrolytes, and creatine-phosphokinase levels were all within the normal ranges. The nerve conduction velocities were markedly decreased. A sural nerve biopsy was carried out and a subtotal reduction in myelin fibers was noted, along with focal endo-neuronal edema. No granuloma formation or inflammatory component could be identified. Based on the clinical, hereditary, and investigative findings, the patient was diagnosed with CMT disease, type 2.

In terms of management, no specific medical treatment is available, but the patient was referred to the orthopedic and rehabilitation departments to manage the foot deformity.

Our second patient, brother of the first patient, was a five-year-old male who presented with difficulty in walking and frequent falls since the past two months. Gradually, his condition had progressed and his distal weakness had worsened, due to which he had been unable to walk without support since the past two weeks. Unlike his brother, the patient could still walk with support, although by dragging his feet. There was no significant difference in the history and examinations of this patient when compared to his brother and similar investigations were carried out, wherein the sural nerve biopsy showed nerve bundles with adequate myelin. Based on the above information, a diagnosis of CMT disease was made here as well and the child was similarly referred to the orthopedic and rehabilitation departments. 

Concerning the apparent autosomal recessive mode of inheritance, the parents of the boys were counseled accordingly regarding the implications of having more children in future and the manner in which their children could further propagate the condition. Additionally, they were counseled to bring in their affected daughters with reportedly similar symptoms for an evaluation and any possible rehabilitation as well, even though the parents described their status as nonambulatory.

## Discussion

Charcot-Marie-Tooth disease, also referred to as hereditary motor and sensory neuropathy (HMSN), is a heterogeneous group of disorders which primarily affects the peripheral nervous system [1]. Clinically, the main features are progressive muscle weakness seen distally, along with wasting seen predominantly in the anterior compartments of the lower legs. Deep tendon reflexes are reduced or absent in most cases and vibratory sense loss, followed by decreased pain and temperature sensations are noted. Foot deformities such as pes cavus and hammer toes are also seen, and most forms of the condition tend to affect males more than females, with the severity of the condition also being increased in males. Research has shown that the disease is mostly inherited in an autosomal dominant pattern. Both our patients displayed most of the clinical features mentioned above [5].

Furthermore, although the disease was earlier classified into two main types, further research has enabled a more detailed classification into CMT type 1, CMT type 2, CMT type 4, and CMTX. These types are then further classified into subtypes [6], with CMT type 1A being the most common variant of the disease [7]. Interestingly, there are several differences between the variants such as the age of presentation and the degree of severity - examples being how individuals suffering from CMT type 2A are nonambulatory before the age of 20 years, while those suffering from CMT type 1A are not and how proximal weakness is encountered sooner in CMT2A, as compared to CMT1A. These differences in characteristics play an essential diagnostic role and further guide investigation [8-9].

Regarding the inheritance pattern of the disease, the autosomal dominant mode is the most common one [10]. A recent study in Japan demonstrated that only 7.2% of the population had inherited the disease in an autosomal recessive pattern which was the mode seen in our case and reportedly, the mode with a more severe phenotype and an earlier onset of symptoms [10-11]. The history of our patients proved to be sufficient when trying to determine the mode of inheritance. In a family of six siblings, four were suffering from similar clinical symptoms, two of whom were females and two were males. Another factor which guided our diagnosis was the fact that both parents were unaffected, but were first degree cousins. This mode of inheritance is reported to occur in less than 10% of cases in Western countries, but in countries where consanguineous marriages are common, such as the Middle East, autosomal recessive CMT accounts 30%-50% of all cases [12].

Mutations in Mitofusin 2 have proven to be the most common cause of CMT disease, type 2A [13]. Moreover, although there are other mutations which can cause CMT type 2, mutations in Mitofusin 2 have been known to cause a severe neuropathy compared to others, with the pattern of the condition being predominantly motor or motor deformity combined with proprioception loss [8]. Most of the mutations result in an autosomal dominant pattern of inheritance [14], but on rare occasion, may be inherited in an autosomal recessive pattern. A study carried out by Polke et al. in 2011 examined three families thoroughly, to determine if the MF2 mutation can lead to CMT type 2 in an autosomal recessive fashion. All relevant investigations including the nerve conduction and genetic studies returned with evidence of the children in the families as having the disease, but most of the parents being unaffected. In the mentioned study, the unaffected parents were heterozygous for the mutation [15].

For the diagnosis of the condition, nerve conduction studies and sural nerve biopsies play pivotal roles. Determining if the type of disease is demyelinating or axonal can be done through nerve conduction studies where, a velocity of more than or equal to 38 m/s characterizes it as axonal, thus making it CMT type 2 disease [5]. A sural nerve biopsy also guides the diagnosis significantly, as it helps in determining if there is any significant demyelination or alternatively, axonal loss - thus differentiating between the two main types, with the former also displaying onion bulb formation. As further progress is made, a newer mode of diagnosis has been reached in the form of genetic testing. Through this method, a diagnosis based on molecular results can be achieved, with the need for sural nerve biopsy diminishing. As nearly 25 genes are said to be involved in the condition, it is no surprise that this newer method of diagnosis is becoming more popular. Another factor which adds to its popularity is how this method is noninvasive, adding to its benefit towards the patient [4]. The management of the condition is mostly supportive, with doctors prescribing physiotherapy and orthoses as effective means of increasing functionality [16-17]. Genetic counseling is a major part of management and both the parents as well as the affected individuals need to be explained the mode of inheritance, so as to prevent the disease from being further passed on. In a society such as ours where consanguineous marriages are the norm, timely counseling becomes even more essential, not just for this disease but for a plethora of rampant inherited conditions [4].

## Conclusions

Thus, we see an uncommon case of autosomal recessive CMT type 2 with its associated clinical presentation, investigative findings, manner of diagnosis and the recommended strategy of management, as well as the general prevalence of the disease, its different classifications, and associated predispositions. This article highlights the need for further cases of the autosomal recessive nature of CMT to be reported; for parents - especially in third world countries - to be counseled to bring in all their affected children for evaluation as well as for large-scale awareness programs and genetic counseling for inhabitants of developing countries, regarding the implications of consanguineous marriages.

## References

[1] Banchs I, Casasnovas C, Albertí A (2009). Diagnosis of Charcot-Marie-Tooth disease. J Biomed Biotechnol.

[2] Vaeth S, Vaeth M, Andersen H, Christensen R, Jensen UB (2017). Charcot-Marie-Tooth disease in Denmark: a nationwide register-based study of mortality, prevalence and incidence. BMJ Open.

[3] Yu Z, Wu X, Xie H (2014). Characteristics of demyelinating Charcot-Marie-Tooth disease with concurrent diabetes mellitus. Int J Clin Exp Pathol.

[4] Szigeti K, Lupski JR (2009). Charcot-Marie-Tooth disease. Eur J Hum Genet.

[5] Harding AE, Thomas PK (1980). The clinical features of hereditary motor and sensory neuropathy types I and II. Brain.

[6] Saporta ASD, Sottile SL, Miller LJ, Feely SM, Siskind CE, Shy ME (2011). Charcot-Marie-Tooth disease subtypes and genetic testing strategies. Ann Neurol.

[7] Wise CA, Garcia CA, Davis SN, Heju Z, Pentao L, Patel PI, Lupski JR (1993). Molecular analyses of unrelated Charcot-Marie-Tooth (CMT) disease patients suggest a high frequency of the CMTIA duplication. Am J Hum Genet.

[8] Feely SM, Laura M, Siskind CE (2011). MFN2 mutations cause severe phenotypes in most patients with CMT2A. Neurology.

[9] Verhamme C, Schaik INV, Koelman JH, Haan RJD, Visser MD (2009). The natural history of Charcot-Marie-Tooth type 1A in adults: a 5-year follow-up study. Brain.

[10] Hoyle JC, Isfort MC, Roggenbuck J, Arnold WD (2015). The genetics of Charcot-Marie-Tooth disease: current trends and future implications for diagnosis and management. Appl Clin Genet.

[11] Yoshimura A, Yuan JH, Hashiguchi A (2019). Genetic profile and onset features of 1005 patients with Charcot-Marie-Tooth disease in Japan. J Neurol Neurosurg Psychiatry.

[12] Patzkó Á, Shy ME (2011). Update on Charcot-Marie-tooth disease. Curr Neurol Neurosci Rep.

[13] Züchner S, Mersiyanova IV, Muglia M (2004). Mutations in the mitochondrial GTPase mitofusin 2 cause Charcot-Marie-Tooth neuropathy type 2A. Nat Genet.

[14] Verhoeven K, Claeys KG, Züchner S (2006). MFN2 mutation distribution and genotype/phenotype correlation in Charcot-Marie-Tooth type 2. Brain.

[15] Polke JM, Laurá M, Pareyson D (2011). Recessive axonal Charcot-Marie-Tooth disease due to compound heterozygous mitofusin 2 mutations. Neurology.

[16] Lindeman E, Spaans F, Reulen J, Leffers P, Drukker J (1999). Progressive resistance training in neuromuscular patients. Effects on force and surface EMG. J Electromyogr Kinesiol.

[17] Mann DC, Hsu JD (1992). Triple arthrodesis in the treatment of fixed cavovarus deformity in adolescent patients with Charcot-Marie-Tooth disease. Foot Ankle.

